# Machine Learning–Based Analysis of Encrypted Medical Data in the Cloud: Qualitative Study of Expert Stakeholders’ Perspectives

**DOI:** 10.2196/21810

**Published:** 2021-09-16

**Authors:** Ala Sarah Alaqra, Bridget Kane, Simone Fischer-Hübner

**Affiliations:** 1 Computer Science and Information Systems Karlstad University Karlstad Sweden; 2 Information Systems Karlstad University Business School Karlstad Sweden; 3 Computer Science Karlstad University Karlstad Sweden

**Keywords:** medical data analysis, encryption, privacy-enhancing technologies, machine learning, stakeholders, tradeoffs, perspectives, eHealth, interviews

## Abstract

**Background:**

Third-party cloud-based data analysis applications are proliferating in electronic health (eHealth) because of the expertise offered and their monetary advantage. However, privacy and security are critical concerns when handling sensitive medical data in the cloud. Technical advances based on “crypto magic” in privacy-preserving machine learning (ML) enable data analysis in encrypted form for maintaining confidentiality. Such privacy-enhancing technologies (PETs) could be counterintuitive to relevant stakeholders in eHealth, which could in turn hinder adoption; thus, more attention is needed on human factors for establishing trust and transparency.

**Objective:**

The aim of this study was to analyze eHealth expert stakeholders’ perspectives and the perceived tradeoffs in regard to data analysis on encrypted medical data in the cloud, and to derive user requirements for development of a privacy-preserving data analysis tool.

**Methods:**

We used semistructured interviews and report on 14 interviews with individuals having medical, technical, or research expertise in eHealth. We used thematic analysis for analyzing interview data. In addition, we conducted a workshop for eliciting requirements.

**Results:**

Our results show differences in the understanding of and in trusting the technology; caution is advised by technical experts, whereas patient safety assurances are required by medical experts. Themes were identified with general perspectives on data privacy and practices (eg, acceptance of using external services), as well as themes highlighting specific perspectives (eg, data protection drawbacks and concerns of the data analysis on encrypted data). The latter themes result in requiring assurances and conformance testing for trusting tools such as the proposed ML-based tool. Communicating privacy, and utility benefits and tradeoffs with stakeholders is essential for trust. Furthermore, stakeholders and their organizations share accountability of patient data. Finally, stakeholders stressed the importance of informing patients about the privacy of their data.

**Conclusions:**

Understanding the benefits and risks of using eHealth PETs is crucial, and collaboration among diverse stakeholders is essential. Assurances of the tool’s privacy, accuracy, and patient safety should be in place for establishing trust of ML-based PETs, especially if used in the cloud.

## Introduction

### Background

Technological applications in health care bring many recognized benefits from providing medical help for remote areas [1], or as a means to tackle medical errors and enhance the quality of medical care [2]. The practice of technology in health care is often referred to as electronic health (eHealth) despite the variety of definitions in applications and research [3].

Machine learning (ML), as a subdomain of artificial intelligence (AI), can be defined as allowing the computer (machine) to learn by finding statistical regularities in data and design algorithms accordingly [4]. ML-based eHealth applications have been emerging recently with the promise of great benefits in the area of medical diagnostics [5]. As ML relies on large datasets, data analysis could be outsourced to the cloud for resource preservation and cost-effectiveness [6]. However, additional privacy and security concerns are raised that need to be addressed by legal and technical measures. Moreover, for establishing end-user trust in ML, data security and privacy are eminent factors [7]. In particular, privacy and security challenges are major concerns that need to be addressed when developing new technologies in eHealth [2,8,9]. Privacy-enhancing technologies (PETs) can help to maintain the functionality of a system while technically protecting/improving the privacy of personal data [10,11].

### Data Protection and eHealth

From the legal perspective, privacy and security regulations differ around the world; various legislations exist for data protection in different jurisdictions. For example, in Canada, the Personal Information Protection and Electronic Documents Act was issued to protect consumers’ data privacy from private businesses [12]. The General Data Protection Regulation (GDPR) in Europe enforces data protection and privacy [13]. In the United States, the Health Insurance Portability and Accountability Act safeguards the privacy and security for medical data specifically [14]. These examples show the different approaches and scopes to regulate the protection, privacy, and security of medical data, which pose a challenge when specifying data-protection mechanisms, apart from geographical jurisdiction considerations.

In eHealth, there exist several strategies that target data protection using the anonymization and deidentification of health data [15]. One example of deidentifying mechanisms for privacy protection is pseudoanonymization, or pseudonymization, where personally identifying data are replaced with pseudonyms to protect a patient’s privacy [16]. Pseudonymization is used for data processing and analysis purposes, where the identity of patients is not needed, and patients can still be reidentified when data are restored to their prepseudonymized state [16]. Conventional medical data protection measures in eHealth, if any are in place, are often not sufficient. There is a recognized need for better approaches to data protection in the medical context [15], such as by deploying PETs.

### Privacy, Security, and Safety Tradeoffs in eHealth

In medical work, the advantage of having records available to several concurrent users over the potential security afforded in a single paper record supports the development of institutional-based electronic health records [17,18]. The contexts where tradeoffs against individual privacy are clear to health care staff include emergency settings, to protect patient safety, or some specific medical contexts. For example, when patient data are being discussed or evaluated between health care professionals, it is part of good communication and practice guidelines that the identity of the person be made known for safety reasons. Communication errors are documented among the leading causes of medical errors [18,19], and the practice of identifying patients correctly helps to reduce medical errors. One would not refer to “the patient in room 53,” “the appendix we had removed yesterday,” or “patient 12345,” for example, because of the potential confusion this could cause that could lead to a medical misadventure. A similar tension among competing interests of protecting privacy, avoiding misleading results, and using data for the public good can be seen in clinical trial data, where protecting patient-level data may compromise the scientific research [20].

However, with the development of cloud and internet services, the risks being taken with respect to preserving private information are not always evident [21]. People have mixed views [22], particularly where medical data are concerned, depending on the context and purpose of use. The existence of the privacy paradox with regard to health-related data is disputed [23]; users do not seem to understand the value of their health data and thus disclose them due to this lack of awareness.

### Analysis on Encrypted Data: Use Case

In this qualitative study, we assessed a privacy-preserving tool that allows automated analysis on encrypted medical data in an untrusted cloud environment. Development of the PET is part of the ongoing EU Horizon2020 research project PAPAYA, which stands for PlAtform for PrivAcY preserving data Analytics [24].

**Figure 1:**
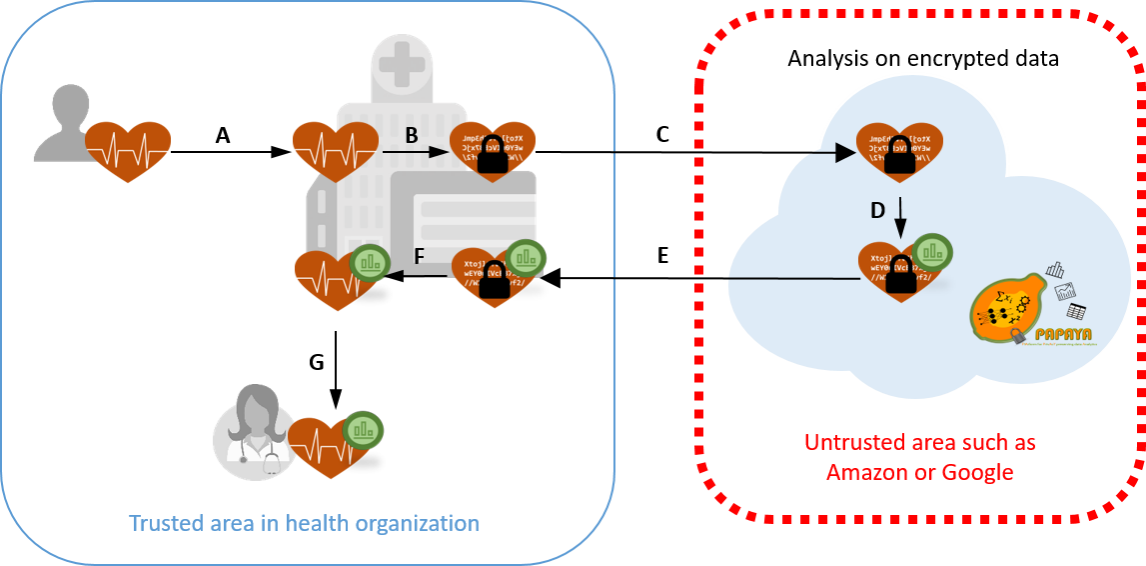
Illustration of use case data analysis flow.

In our interviews, as part of the PAPAYA project, we focused on an eHealth use case related to analysis of electrocardiogram (ECG) data. In the use case scenario, the patient needs to perform cardiac function analysis for a heart-related diagnosis. For this purpose, the patient wears a sensor device that they obtain from a pharmacy to collect their ECG signal data for a period of 24 hours. Upon returning the device to the pharmacy, the data are downloaded and transferred to a medical health platform (Figure 1A), where the ECG signal data are then encrypted (Figure 1B). The encrypted data are then submitted to a data analysis platform running in an untrusted cloud environment (Figure 1C). The data are then automatically analyzed on the PAPAYA platform (Figure 1D). For protecting the patient’s privacy, a privacy-enhancing ML tool (PAPAYA tool) is used on the data analysis platform (PAPAYA platform). Hereafter, we use the acronym PAPAYA to refer to the PAPAYA tool running on the PAPAYA platform. The neural network model used for data classification is executed over encrypted data by utilizing advanced cryptographic schemes such as homomorphic encryption [25] or secure multiparty computation [26-28]. The encrypted automatic analysis report is sent back to the medical health platform (Figure 1E), where it is decrypted (Figure 1F) and then forwarded in plain (ie, unencrypted) form to a cardiologist together with the raw ECG signal data (Figure 1G). The cardiologist then uses both inputs to produce a report on the patient’s heart status.

The data analysis tool running on the PAPAYA platform in the cloud has no user interfaces to be used by doctors or patients. However, the analysis report sent to the patient can be displayed via a dedicated dashboard.

Many PETs for protecting and anonymizing medical data or medical data analyses are based on data generalization or adding statistical “noise,” and thus a tradeoff between privacy protection and data quality is required. In contrast to these types of PETs, the privacy-preserving tool that is the subject of this study uses cryptographic approaches that do not affect the quality of the analysis result; however, this may not be obvious to stakeholders or users.

Establishing trust is an important component for acceptability and the adoption of technology [29,30], and is especially a challenge for the proposed PET based on “crypto magic,” which may be counterintuitive for stakeholders in eHealth. The privacy and security properties of a PET based on analysis of encrypted data in the cloud may not be perceived correctly. Therefore, we focused on human factors and investigated user requirements in terms of measures for establishing trust and transparency for the relevant stakeholders.

### Research Objective

The objective of this study was to analyze eHealth stakeholders’ perspectives and the perceived tradeoffs concerning data analysis on encrypted medical data in the cloud. Additionally, we aimed to derive user requirements for the upcoming development of the privacy-preserving data analysis tool and its dashboard (ie, the interfaces for viewing data analysis by the cardiologist). Therefore, our research questions were as follows: (1) What are the perspectives, understandings, and privacy concerns regarding the analysis of encrypted medical data in the cloud of eHealth expert stakeholders having medical, technical, and research expertise? (2) What are the user requirements for the development of privacy-preserving data analysis tools based on ML?

A user-centric approach has been advocated to be of importance in the area of privacy and data management [31]. This study explored perspectives from eHealth stakeholders varying in their expertise (medical, technical, and research) involved in medical data analysis. By reporting on the insights of the stakeholders in this study, and identifying requirements, challenges, and perceived tradeoffs, we can contribute to advancing the state of the art of human factors related to the analysis of encrypted medical data in an untrusted environment (cloud). The investigation of human-computer interaction (HCI)-related issues with regard to protecting privacy in ML and the analysis on encrypted data, particularly in health care, is a main novel aspect of this study.

## Methods

### Design

Since the analysis on encrypted medical data in the cloud is a novel application in the medical area, our user-centered design approach focuses on the user’s perspectives and concerns regarding the applicability and acceptability of the given PET. We therefore followed an exploratory approach, using empirical qualitative means for our data collection to understand stakeholders’ perspectives and concerns regarding the analysis of encrypted medical data in the cloud. Qualitative methods allow for in-depth investigation of participants’ understanding and perspectives, which is crucial when it comes to investigating a concept in its development stages [32].

To investigate the perspectives, understanding, and privacy concerns of expert stakeholders in eHealth of the tool for the privacy-preserving analysis on encrypted medical data, we chose semistructured interviews. To elicit user requirements from the interviews, we conducted a workshop.

### Interview Structure

We chose semistructured interviews as our method of investigation, which allows for flexibility while maintaining some key concepts to be covered in the discussion. We interviewed stakeholders who have knowledge related to medical data analysis of the ECG test. The interviews allowed one-on-one conversation with each interviewee to gain their in-depth perspective on the matter. The semistructured form offered the flexibility to investigate parallel subtopics of the different stakeholders’ expertise: medical, technical, and research.

In our interviews, we had general questions inquiring about the participant’s background and privacy routines, followed by an introduction to the use case and specific questions about the analysis of encrypted ECG data (see Multimedia Appendix 1). Since our participants had diverse expertise, it was relevant for our study to understand the context of their privacy practices and opinions in general before we discussed the specifics of our PET tool.

### Recruitment and Sampling

The study consisted of 14 interviews in total with stakeholders of varying expertise. Initially, we targeted medical professionals for our interviews since we were interested in understanding any medical concerns in performing data analysis on encrypted medical data. However, we expanded our recruitment to include those with technical expertise and researchers in the area of eHealth owing to their involvement with medical data processes (as highlighted by initial interviews with medical experts). Our inclusion criteria included being familiar with the ECG test and analysis on medical data. We deliberately did not include any interviewees that were affiliated with the PAPAYA project partners, as this could have introduced a bias. Hence, none of the interviewees had heard about the PAPAYA project prior to the interviews. Eventually, following purposive sampling, we recruited 14 individuals and satisfied our data saturation. Table 1 provides details on the interviewee identifiers with their corresponding expertise (eg, Med1 stands for interviewee #1 with medical expertise). Our interviewees represent a cross-section of experience and specialized knowledge that is typically encountered in medical work. We report the participants’ demographics in aggregated form in consideration of our ethical responsibility to preserve participant anonymity.

This study was performed with participants from different countries, age groups, and genders, allowing our sample to be diverse with regard to the inclusion criteria. Using our own professional networks and those of our project partner Media Clinics Italy, we selected experts based on their expertise and knowledge of ECG in Sweden (n=4), Italy (n=2), the United Kingdom (n=2), Ireland (n=4), and Australia (n=2) for purposeful sampling, and recruited the participants via personal invitations for the interview. The 14 interviewees were drawn to investigate any preliminary differences in regulations. The male:female ratio was 5:2. They reported their age in the range of 21-30 (n=2), 41-50 (n=4), 51-60 (n=4), and ≥61 (n=3) years, and one participant chose not to disclose their age group. All participants worked in a public organization either full or part time, apart from one participant who worked in a semigovernmental organization. The experience of the medical professionals varied from 5 years to more than 30 years, whereas the experience of researchers and technical experts varied from 3 years to over 29 years.

**Table 1:** Interviewee index with their corresponding expertise details.

Interviewee	Expertise	Details
Med1	Medical	Nurse in cardiothoracic care
Med2	Medical	Director of care center, with nursing experience
Res3	Research	Chief information officer in health informatics
MedRes4	Medical+research	Emergency physician with academic posts in medical informatics
Med5	Medical	Family doctor
Med6	Medical	Medical doctor with urology expertise
Res7	Research	Health systems research leader
ResTec8	Research+technical	Professor with computing experience in digital health
MedRes9	Medical+research	Primary physician and professor in informatics and electronic health
MedRes10	Medical+research	Medical doctor and researcher consultant in cardiovascular surgery
Tec11	Technical	Information technology security manager
Res12	Research	Researcher in public health
Med13	Medical	Medical doctor and trainee anesthetist
ResTec14	Research+technical	Researcher in electronic health and cybersecurity

### Data Collection

To adhere to the differences in our stakeholders’ expertise, we followed a flexible approach at each interview using a semistructured format. Additionally, since our participants were situated in different locations around the world, we used an online meeting tool for the video call in addition to sharing screens. All interviews were conducted online using the online GotoMeeting tool [33], except for two interviews that were able to be conducted face-to-face. The interviews lasted 30-60 minutes, depending on the expertise of participants (ie, those with technical expertise were able to discuss further technical questions). All interviewees, except for one, consented to their interview being recorded. There were 2-3 interviewers who are privacy and HCI researchers with technical, HCI, and medical expertise present in all interviews, with one leading the interview while the others took notes and added follow-up questions. The data collected are based on the combined notes. In case of conflicts in notes, we included results that were either resolved by our workshops or by referring to the recordings; otherwise, such results were not considered. All interviews, except one, were conducted in English; the exception was an interview that was conducted in Italian in Italy with the aid of translators and collaborators from the project partners.

Interviewees were provided with the consent form and introduction to the study prior to the interview. An interview guide was used by the interviewers, as found in Multimedia Appendix 1. During the interview, participants were given a short introduction followed by introductory questions targeting their background experience, and their understanding of protection needs and the privacy routines practiced in their organizations. The introductory questions allowed us to understand their current situation and better understand their perspectives of the next sections. The use case was then introduced using presentation slides, followed by questions about their perception of privacy and trust, and privacy protection relating to the use case. Media Clinics Italia, our project collaborators who are implementing the use case application, provided us with the presentation slides introducing the use case and the functionality of the PAPAYA platform. The slides include the use case description and correspond mostly to the description that we provide above in the “Analysis on Encrypted Data: Use Case” subsection, presenting the actors and data items involved, as well as a high-level presentation of the use data flow (deconstructed versions of Figure 1). However, no details on the encryption algorithms were provided to the interviewees. Instead, it was only conveyed that the ECG data are analyzed by the PAPAYA platform in encrypted form and that the output in form of the analysis report is also encrypted.

**Figure 2:**
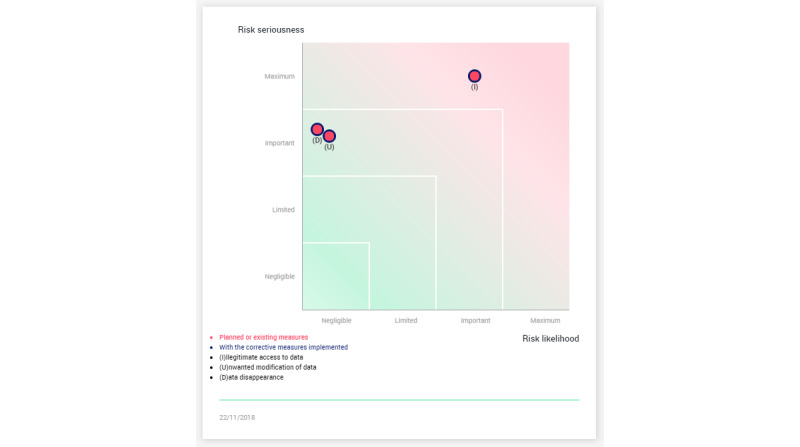
Risk assessment without PAPAYA. PAPAYA: PlAtform for PrivAcY preserving data Analytics.

**Figure 3:**
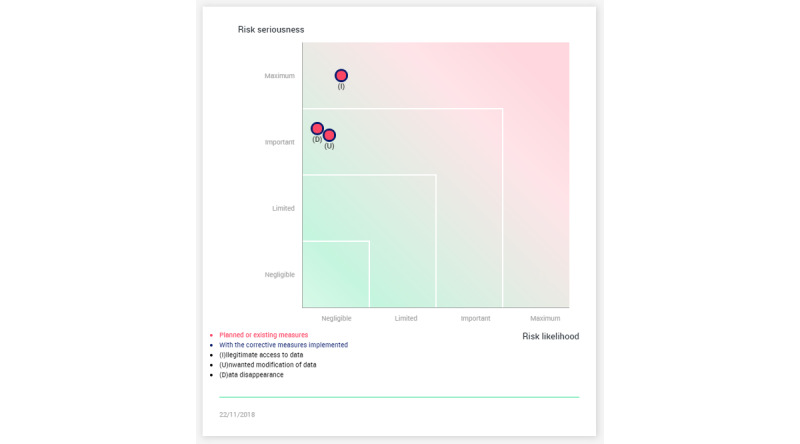
Risk assessment with PAPAYA. PAPAYA: PlAtform for PrivAcY preserving data Analytics.

Questions about their trust of the tool and accountability followed showing figures on privacy risk assessment with (Figure 2) and without (Figure 3) using PAPAYA. The provided privacy impact assessment (PIA) was a result of using a PIA tool developed by the French data protection authority Commission Nationale de l'Informatique et des Libertés [34]. Finally, questions regarding informing patients and the level of knowledge needed about the platform were asked.

Between interviews, the three experts met to discuss and analyze the progress of the interviews. Additionally, they discussed whom to recruit next, depending on the expertise needed for the study. Participants continued to be recruited until data saturation was reached in our investigation for each of the expertise groups (medical, technical, and research).

### Data Analysis

We used the data from our interviews for two purposes: the first was to analyze the data to gain an understanding of the participants’ perspectives, and the second was to derive user requirements for application implications.

Since our study is qualitative, we have generally looked for patterns and incidents of participants’ responses, and then classified our data into categories/themes. We used the structured approach for coding and categorizing our data that falls under the umbrella of the analytical method known as thematic analysis [35,36]. Since thematic analysis is a rather flexible approach [37], we explain our analytical process in detail below.

There were three phases to the data analysis procedure to find patterns and categorize our results into themes. The first phase was to have an agreed-upon results record for each interview. Apart from note-taking during the interviews, the three interviewers independently reviewed the recordings and elaborated on their personal notes (for reliability purposes, added confidence notes on interviewees’ responses) they had each taken, using the structure of the interview guide as a template for consistency. They met afterward in a session to discuss the interviews and verify the meanings of the responses; they then merged their notes into an agreed-upon record for each interview. Conflicts and issues that were raised or observed at a particular interview were considered by a further review of the recordings of the interview to double check if there was any omission from the individual interview record. Any discrepancies were reviewed independently with the original digital recording and later rediscussed in a meeting. The second phase involved two rounds of coding of the results to reach an agreement on the codebook. The interviewers independently coded the results record, and discussed the codes together afterward with initial themes and formation of topologies. The third phase involved summarizing and synthesizing the data with the finalized themes. The interviewers met in a session to discuss and evaluate the themes developed and refined in the analysis. Since the interviews were semistructured, the analysis was not purely deductive, since the structure of the interview guide and the questions posed to the interviewees were used in the analysis.

### Requirements Elicitation Workshop

To elicit user requirements for addressing our second research question, we conducted a workshop with the three researchers who were taking part in the interviews. The workshop was established to discuss and elicit requirements from the results of the interviews, while taking into consideration legal and technical aspects of the privacy-preserving ML technology. The format of the workshop allowed for metalayered discussion of requirements stated by the interviewees and critique of our results. In addition, the workshop discussion focused on some general implications for both research and practice for future ML applications in eHealth.

### Ethical Considerations

The study was reviewed by the ethical advisor of Karlstad University, along with review of the provided interview guide and consent form. The advisor officially confirmed, according to the national regulations, that there are no ethical concerns and no further ethical review was needed by the Institutional Review Board according to the Swedish National Ethics Review Act. Participation in the study was voluntary, and the recording of the session as well as the demographic questions were all optional. No sensitive data were collected, and participants were instructed to not reveal sensitive data (eg, any information related to their own health). They were explicitly instructed at the beginning of the interview that in the case of this happening, the recording would be stopped to remove that part of the interview. The consent form given to interviewees prior to the interviews contained the above information.

## Results

### Interviews

#### Overall Themes

We present key results from our thematic analysis of the interviews, categorized in the following subsections. Summary detail on our analysis is provided in Multimedia Appendix 2.

#### Contextual Protection of ECG Data

When discussing the level needed for protecting ECG data, pseudonymization and encryption were the main approaches mentioned. As emphasized by the interviewees, all patient information is treated in the same way: as private and confidential, and national regulations are taken into consideration. Overall, protection of ECG data in terms of pseudonymization and other current measures is regarded as sufficient depending on the context of where the data are and how they are processed; however, further protection is needed when data are transferred to external processors and especially when using the cloud.

Several participants (Med1, Med2, MedRes4, Med6, Res7) indicated that they do not have the technical expertise to be able to identify what is needed in terms of data protection and encryption; for instance, Med1 stated that it is “…a question for IT (information technology),” Med6 stated that “…would need a computer scientist to answer,” and Res7 mentioned “I am not a technical person,” and that it is the responsibility of their engineer. They consider the responsibility to be that of the information technology specialists or data analysts. Most participants claimed that privacy can be better protected if the identifying data are kept separately from the signal data (pseudonymized). However, the issue of data integrity was raised if the signals are to be stored separately from the metadata.

A distinction should be made between reading the ECG and interpretation of the result in context. Medical expertise participants expressed the need to review the individual ECG results as well as the medical record when making a diagnosis and treating their patient:

On its own an ECG is not worth much…to give an opinion one needs the history, as well as ethnicity and age…you need to know why they are concerned and if they have a family history…[Med1]

Data privacy measures in clinics usually start with staff induction regarding security and privacy. Health professionals are taught about managing a patient’s chart, policies, and guidelines (ie, maintaining the integrity of records). Participants spoke of protecting patient privacy through physical separation in space, locked files, storing data with access control, two-factor authentication (with smart cards in combination with passwords), firewalls, network segmentation, encrypted USB keys, and using secure devices and computers.

Electronic access is normally controlled, and all interactions are logged in a file to which the patient can have access. Auditing of interactions is regularly performed. In some cases, security is contracted to an external company (MedRes4).

Participants reported that pseudonymization is considered satisfactory to protect an individual’s privacy when performing research using ECG traces without patient-identifying information (Res7, Res12, ResTech14). Guidelines from ethical committees and research approvals are sufficient for research involving medical data.

However, especially in the cases of transferring data outside the organization, participants indicated that pseudonymization does not offer sufficient protection for an individual; the ECG needs to be encrypted if transmitted or stored in the cloud (Res3, Med5, MedRes9, Tech11). Tech11 pointed out that ECG data are stored on the physical ECG machine and are not protected; thus, these data are exposed to “privacy leaks” in case of active adversary attempts, which should be considered. It was also highlighted by Med1, Med2, Res3, Med6, Res12, and Med13 that to maintain the trust of the public, one must ensure protection against privacy breaches of medical data. Two participants referred to prior incidents where data had been leaked when storing at external servers (Med2, MedRes10).

#### Conditional Sensitivity of ECG Data

The ECG test is considered medical data, having the same status as other medical information, and is thus considered private and confidential. Almost all participants regarded the ECG test results as sensitive, because they constitute medical data and therefore by default are sensitive. Many also referred to the legal regulations as the guide for indicating the sensitivity of medical data.

In the discussion on whether the raw data of the ECG, apart from being medical data, are sensitive, only MedRes4 and MedRes9 stated that ECG data could still be considered sensitive despite pseudonymization, and therefore need protection.

Many participants pointed out that the sensitivity of data also depends on the other information they are combined with, and the additional associations that are made (Med1, Res12, Med13, ResTec14). For example, the very fact that someone had reason to have an ECG is considered private information, as the ECG can demonstrate heart disease. Moreover, it was stated that it can be compromising for a patient if an employer or insurance company learns that they underwent an ECG test. For example, professional athletes could have their career destroyed if they were known to have an abnormal ECG.

Additionally, most participants do not regard the ECG to be as “sensitive” as some other tests. With regard to ECG data, MedRes4 stated that “it is clinical data…all information about the patient is sensitive,” and that blood tests, or having it known that one tested, for “having cocaine…is more sensitive.” For instance, Med2 ranked the sensitivity of ECG data “on a scale 1 to 10, probably about a 6.”

A distinction was made between data that are gathered and given voluntarily by the individual via personal devices and data that are gathered by a health professional (ResTec8, Res3). It was argued that the ECG data, if supplied by the individual, are considered nonsensitive, because these are consumer-contributed data; however, if taken by a medical professional, these data are considered to be sensitive (Res3).

#### Acceptance of Using External Services

When discussing engaging external services for managing the privacy and security of their data, most regarded it to be impossible to provide a guarantee without any remaining risk to individual privacy. They also accept a tradeoff between risks and benefits, and indicated that some risk might be acceptable if the service is proven to be valuable. Few participants indicated that they would use external services for conducting medical and research trials (Med6), in case of necessity (Res3), or depending on the organization’s policy (ResTech8). However, they indicated a need to weigh the pros and cons before making such a move.

Many reported that they already use external services for either outsourcing ECG (Tech11), managing security (Med13, ResTech14), managing computers (Med1), and storage (Med5). Res7 and Med2 reported that they already work with cloud services (such as Amazon), where security and testing are requirements and the use of the cloud service is cautiously accepted, provided that privacy is protected and security is adequately assured by a qualified entity. Med2 added that “working with cloud computing and services, we can’t be masters of everything.” However, concerns and skepticism were highlighted regarding the lack of trust in cloud services, and several participants (MedRes10, Res12, ResTech14) stated that they prefer using internal mechanisms within their organization. MedRes10 added:

if you have a company which doesn’t understand why it is important to have patient’s secrecy, then actually patient’s data can be leaked out…the information we receive at the hospital is important information for companies and if you have a leak at such a source (external), that can be very important for commercial companies.

#### Data Protection Drawbacks

Pseudonymization may sometimes be used to avoid sharing sensitive information within a circle, such as when asking for informal advice on a case between colleagues. However, pseudonymization is not very commonly used in clinical practice, except in research contexts, as indicated by the participants (Med6, Res7, ResTech8, MedRes9, and ResTech14) and for lab tests. When discussing data protection, Res3 stated that “data protection does not trump everything…not so black and white”; thus, one needs to weigh the risks and benefits of a technology, effects on safety, and perception by human factors.

For most nontechnical participants, there was a common belief that encryption is being performed by the institution behind the scenes. For example, encrypted email is common within their organization. Encrypted data are better protected than unencrypted data. Encryption is considered important, especially for data that are being transferred between institutions. It was mentioned that encrypted data are considered to be safer than unencrypted data; however, encryption “cannot be trusted 100%” (Res12). Med2, Med5, and ResTech14 expressed concerns about data being encrypted, wherein Med2 and ResTech14 stated that too much encryption has a risk of corrupting data and may result in loss of data integrity.

You have to balance the risks with the actual report of the true data,…as a rule of thumb, if we can encrypt without impacting the message in a negative way then it is worth doing…if you start to do a value risk on this, it may be too much encryption…we want the most effective and true result as we can get.

Med2

Some incidents were mentioned by Med2 and ResTech14 where data loss occurred due to encryption; cryptographic solutions introduced without proper testing resulted in loss of data. It was highlighted that complete and available data have higher priority in this case.

#### Concerns of Data Analysis on Encrypted Data

The proposed analysis on encrypted data was well-received by some participants (Med6, Res7, Med13), who believed that they would trust the analysis given proper testing, proofs, and validation studies are provided. Res7 added, “it depends on who did the algorithm behind it…it surely must be tested.” Moreover, Med1 and MedRes10 expressed the need for other sources of data in combination to trust the result. The above-mentioned perspectives highlight the *necessities for acceptance* of the proposed technology as expressed by the participants.

However, others expressed strong doubts regarding the algorithm used (ResTech8, ResTech14) and data accuracy resulting from the analysis (Med5). Doubts about the technical possibility were emphasized by MedRes4 and MedRes9. MedRes4 stated, “I didn’t know it [analysis of encrypted data] is technically possible...I need to check that out…[encryption is] changing all the time.” MedRes9 responded with, “Sorry, sounds like bull**** to me…I don’t believe that” regarding the analysis on encrypted data, and added:

to analyze ECG while they are being encrypted doesn’t make sense… it totally depends on what encryption scheme…You can imagine some simple encryption scheme, might be possible to analyze some aspects… but in the more general sense it’s nearly impossible. It’s like analyzing a picture which is encrypted, how would you do that?

Other medical participants remain skeptical that an analysis could be performed on encrypted data and query if only the identifying data are encrypted rather than the raw signal data. Although its proposed use as a screening tool was explained, some medical participants expressed concerns regarding the motivation for using the tool without a cardiologist’s guidance, and that this method might be (wrongly) used alone to diagnose a heart disease. Furthermore, Tech11 stated that medical expertise is needed for determining the accuracy of the ECG. By contrast, Res12 stated that technical expertise is needed to answer for the accuracy of the data analysis.

#### Communicating Privacy and Utility Benefits and Tradeoffs

When discussing trust in the data analysis service, we investigated participants’ opinions on providing two different trust statements. The first statement was: “The patient’s data will be analyzed in encrypted form so that private data cannot leak to the PAPAYA analytics service; this form of analysis will not negatively impact the data quality.” Participants expressed doubts regarding the technological plausibility (MedRes4 and MedRes9) and the encryption (Tech11 and ResTech14). MedRes4 further stated that “would need expert opinion…and contact data-analyst experts.”

Participants discussed trust in terms of what is needed, namely the *information* and *reassurances* for trust.

They expressed that trust is dependent on information about the level of protection (Med6), trusting the company of the technology (Med5), or trusting the tool (Res7, Med13). Res12 and ResTech8 expressed the need for more information overall. Specifically, ResTech8 expressed a distrust of the statement:

it doesn’t tell me anything about how it’s going to be encrypted, what other forms of prevention of leakage might occur, what kind of analytics are going to be undertaken…all of those affect my ability to trust and retain privacy…as a statement of fact it is not believable, as a statement of intention it is believable.

We also inquired about the significance of assurances on trusting the tool. In our interviews, we provided an example of the case where an organization would state that they conducted a PIA. We asked the interviewees the extent to which they would trust PAPAYA if it was stated that this PIA would show a risk reduction for illegitimate access to data from important (Figure 2) to negligible (Figure 3) when using PAPAYA. Some participants (MedRes10, Tec11, Med2) noted the fact that an organization that made the effort to conduct a PIA would generally increase their trust in PAPAYA or that the PIA could be useful to convince decision makers (ResTec14). Med5 considered that the statement was useful and no further information was needed.

However, other participants discussed requests for PIA (privacy and utility benefits) *assurances.* They wanted to have more information about the PIA method (Tec11), how the PIA was conducted (eg, MedRes9 stated, “I would need more detailed descriptions on how they arrive to these measurements…not just presenting them on a diagram”), and about the qualification of the individuals that conducted the PIA (ResTec8) in order to trust the statement. Participants highlighted the need for validation and testing of the tool (Med1, Med2, Res3, MedRes10, Tech11), as well as certifications (Med2, ResTech8, MedTech10, Tech11, ResTech14). Moreover, Res3 highlighted the need for risk assessment, and that tradeoffs between safety and data protection should be addressed; more information should be provided on data quality and costs.

#### Shared Responsibility for Patient Privacy

When discussing responsibility for patients’ privacy, participants indicated that they share responsibility with the organization in this regard (Med1, Res3, MedRes4, Med6, ResTech8, MedRes10, Tech11, Med13). Medical registration depends on observing codes of conduct to protect the patient. However, the institutions employing the professionals (and researchers) have a legal responsibility in most jurisdictions, and accountability rests with the chief operating officer, chairman of the procurement group, or head medical person. In research, the principal investigator is normally the person held accountable for any data breaches (MedRes4, Res7, Res12, ResTech14). Med2 mentioned that “we are bound…by our scope of practice in nursing, with a strong approach to managing patients’ confidentiality,” and stated that “if it is a data breach from a system perspective, then I think it comes back to the organization.” A bigger proportion of responsibility for data protection and security was considered to rest on the organization’s security team through technically securing the data; however, nontechnical participants indicated that they do have a say in the applications used and technical infrastructure.

#### Informing Patients on a Higher Level of Abstraction

The majority of participants do not expect to know in expert detail on how the privacy measures are in place; they want to have sufficient knowledge to be able to explain how the data are used and where they are stored. However, all participants stressed the importance of having information available to all patients. They (Med1, MedRes4, Med5. Med6, Res7, MedRes9, Med13, ResTech14) argued that it is especially important to be able to provide information if people ask for it, by being able to refer to an expert in addition to offering handouts (eg, leaflets).

Informing patients proactively about their rights to privacy and how their data are being protected was perceived as essential by Med2 and MedRes10. However, others suggested that trusting the organization, health systems, and health professionals is sufficient (ResTech8), and trusting that privacy measures are in place has higher priority (Res3). For example, ResTech8 pointed out that according to the national digital health agency,

probably 5% would be interested to know and would seek to know…and probably another maybe 10% of patients, if you told them, they would in retrospective be interested in knowing...and the remaining 85% of patients would be uninterested…they would just trust the health system.

Med5, Med6, and Tech11 indicated that basic knowledge is sufficient to inform patients about the current measures in place to ensure privacy and protection of their data.

### Workshop Requirements

Based on our interviews, the following key requirements were elicited relating to perceived tradeoffs and perceptions on informing eHealth stakeholders and patients about the proposed PET (as developed by the PAPAYA project), and its security and privacy protection features for enhancing transparency and promoting trust. Further details on user requirements and legal requirements elicited can be found in our prior work [38]. Notably, the following requirements are not specific to PAPAYA, but are in fact generalizable to similar PETs for automated data analysis of medical data on a cloud server.

First, eHealth stakeholders will be reluctant to avail of the analytical services in the cloud if they have no confidence that the PET can deliver secure service without loss of quality or data. Therefore, reassurances are required for trusting the proposed PET by providing assurance guarantees confirming that analysis on encrypted data on the privacy-preserving data analytics platform was validated and certified to work as stated to the stakeholders, and making the reports of conformance tests of the platform available. Second, results from a PIA conducted by qualified experts should be presented to all stakeholders for communicating privacy benefits and tradeoffs, comparing the situations when the PET is used or not used. These results should be complemented with information about the PIA evaluation method, process, and qualification of the evaluator. Having information on a PIA available shows that the service provider takes privacy seriously, which can aid users in making decisions on tradeoffs between benefits and privacy risks. Third, stakeholders have indicated the importance of providing information regarding data protection and privacy of their data, and that transparency to patients is crucial. Hence, it is important that medical doctors can address privacy-related questions from the patient side by informing them about privacy protection and data quality guarantees via leaflets or tutorials. Lastly, as suggested by study participants, interested patients should be informed proactively about their rights to privacy and how their data are being protected at the moment when they are requested to provide consent.

## Discussion

### Principal Findings

To better understand eHealth stakeholders’ perspectives, knowledge, and privacy concerns regarding analysis of encrypted medical data in the cloud (our first research question), our results from the interviews brought forth themes that correspond to the general stakeholders’ perspectives on data privacy and practices. These themes include (1) *contextual protection of the ECG data*, (2) *conditional sensitivity of ECG data*, and (3) *acceptance of using external services*.

Furthermore, our themes highlight (4) *data protection drawbacks* in general and (5) concerns of *data analysis on encrypted data specific to an ML-based tool*.

Trusting data and the technology is essential, which is achieved by (6) *communicating privacy and utility benefits and tradeoffs*. In addition, accountability is important, and the participants highlighted that there is (7) *a shared responsibility for patient privacy*. Furthermore, when it comes to accessibility of information about the technology used and how data are managed, (8) *informing patients on a higher level of abstraction* was emphasized.

Finally, our workshop derived user requirements for the data analysis on encrypted data in the cloud, which are generalizable to similar ML applications, thereby addressing our second research question (to establish user requirements).

### Related Work on Privacy and ECG Data Analysis

Earlier research on remote cardiac monitoring in hospitals or with telemedicine proposed plain processing of the ECG data based on the most common parameters such as cycle length variability (RR intervals) [39,40], whereas more recent work has applied modern techniques based on ML to perform more structured analyses [41]. As might be expected in this type of research, attention tends to be focused on analytical methods to the signal rather than to appreciate the sensitivity of these health data and the concern for privacy. Thus, there is earlier research on the analysis of ECG data without regard for how privacy can be protected. Some more recent studies propose applying encryption to data prior to the data being outsourced for analysis [42]; however, when ML is considered for ECG analysis, attention to privacy diminishes. For example, Kocabas and Soyata [43] applied full homomorphic encryption on ECG data for analysis in a public cloud; however, neither legal privacy nor user requirements were discussed.

We previously reported the PAPAYA arrhythmia detection use case, and legal and user requirements [38]; however, we did not elaborate on the analysis of the eHealth stakeholders’ perspectives and the perceived tradeoffs based on the conducted interviews.

### Biometric ECG and Data Protection

Previous studies have focused on enhancing the privacy of ECG data using cryptographic schemes [42,44]. However, in this study, we focused on human aspects and involved stakeholders’ perspectives on the proposed privacy-preserving solution. A significant outcome of our study is the perception of data sensitivity and data protection by participants. Apart from legal aspects, where the majority of our participants considered medical data sensitive by default (referring to laws on medical data privacy), most expressed the view that the ECG signal is not a personally identifying measurement or biometric, and that pseudonymization should be sufficient. Only two participants (with medical/research expertise) had a different perspective, and argued that the ECG signal is sensitive despite pseudonymization. However, it has been shown that raw ECG signal data are indeed biometric data and thus, even if pseudonymized, they classify as personally identifiable data [45].

Medical data are classified under a special category of data according to Article 9 of the GDPR [46], and thus require special protection. Similarly, participants who regarded ECG data as nonidentifiable data (nonbiometric) still expressed the view that ECG as medical data are sensitive data, and thus require special protection. Therefore, they consider that medical data should be protected in any case, even if it is claimed that the data are anonymized. Hence, the participants are aware of the required protection (eg, as in our use case via encryption), even though the legal and technical reasons for the protection may not be fully understood.

### Expertise Differences and Collaboration

Previous studies exploring human factors, perceptions, and trust of PETs show differences in trusting PETs and tools; those with more technical expertise, except for crypto experts, would require more information to trust the tools, which are often based on nonintuitive “crypto magic” operations [47,48]. Similarly in this study, depending on the background of the experts (whether technical or medical), the trust criteria required for this technology differed. Participants with research and technical expertise expressed significant concern for trusting the feasibility of the technology and algorithms, whereas data availability appeared to be more important to the medical experts in general. It is noted that perspectives of participants and their expertise were conflicting when discussing data accuracy: medical experts highlighted the need for technical experts to answer for data accuracy of the tool, whereas technical experts stated that medical opinion on validation is needed. Collaboration among computer scientists and physicians is not new; the focus on different values has been shown to be fruitful [49]. Therefore, there is a clear need for communication and collaboration among different stakeholders with different expertise in eHealth.

### Privacy Tradeoffs in eHealth and Trust Assurance for PETs

Previous studies have followed different approaches in dealing with privacy, security and safety tradeoffs, and challenges in eHealth [50-52], including balancing tradeoffs between privacy protection and information utilization in eHealth [51], information accountability [52], or risk mitigation management processes [50].

In our study, when discussing tradeoffs involved in the data analysis tool, functionality, accuracy, and data availability emerged as the main tradeoffs with respect to discussions on privacy and security. It is clear that if security and privacy schemes would hinder the availability of data or corrupt the data, then it is not worth the risk. Trust criteria were key factors in the discussion (eg, trusting the functionality, availability, encryption, organization, or the tool). For instance, having certifications by third parties has been shown to enhance trust [53,54]. Therefore, we argue that in the case of PETs in eHealth, trust assurances should be provided relating to the availability of data so that the safety of patients is ensured.

### Trust Assurance for ML

Previous studies have addressed physicians’ perspectives on ML tools and trusting the outcomes, showing that physicians desire to understand the logic of the ML tool in order to trust the results [55-57]. In a more recent study that focused only on clinicians’ perspectives in an ML-based AI system, trust optimization was key in addressing the adoption of the technologies [58]. Challenges for trust in medical AI by the public; the role of credibility of technology companies; as well as the need of transparency, certification, and education for medical AI have all been described [53,54].

With regard to trust in privacy-preserving ML, as the focus of our work, trust issues may arise on the privacy-preserving crypto algorithms concerning functionality, data accuracy, and availability (see above) on top of general trust issues that may already exist in regard to ML. Thus, the requirement for trust assurance for privacy-preserving ML, as stated above based on our finding, is especially relevant.

### Education and Information

Challen et al [55] argued that the medical education curriculum should train medical professionals adequately in AI, including ML, along with its advantages, including improvement of quality, and shortfalls such as transparency and liability. Based on our findings, we suggest that such training should also teach medical professionals about PETs for ML to increase their trust, knowledge, and competence for informing interested patients and answering their privacy-related queries.

Support for patients concerning explanations regarding the technologies used, how they might be affected, and informed consent have also been reported to be important for trust, since patients are usually unfamiliar with the technologies used in eHealth and may not be convinced with the benefits of using such technologies [59]. We previously discussed user perceptions and requirements for other types of novel privacy-enhancing eHealth use cases [47], which, in line with this study, showed that even users with more technical expertise also require information about assurance guarantees to trust the claimed privacy-preserving properties of the technology.

For developing usable consent forms that clearly convey the core policy information to all types of users, such technical information should be easily retrievable via clickable links upon demand by interested users rather than the detail shown by default. Therefore, we propose following the suggestion in Article 29 Working Party [60] for using layered privacy notices, which make technical information about privacy protection accessible at lower layers with different layers of details.

### Implications and Future Studies

Our work dealing with a privacy-preserving ML tool and its application in the ECG use case has focused on the human aspects from stakeholders’ perspectives on the expert side (medical, technical, and research expertise). Our contribution highlights key areas (themes) and requirements for future applications of the dashboard for the tool as well as user-centered research in eHealth of ML, and especially research on the effectiveness of means for trust assurance (eg, via clear communication, certification of PETs for ML, and education of medical professionals). Although our study focused on the perspectives of eHealth professionals, future studies should also investigate patients’ perspectives and trust criteria for having their data used by new ML-based technologies.

### Limitations

Given the relatively small number of participants per category, it is not possible to make definitive claims regarding their countries, gender, or age. However, we have included a diverse sample in our exploration of possible concerns and requirements for the PETs addressed. Additionally, due to our selection criteria of our target group, it was challenging to recruit stakeholders given their demanding professions and limited availability. Further studies could explore if there are general trends to be noted.

### Conclusions

Understanding the benefits and risks of using ML-based analysis of encrypted medical data is crucial. Interviewing stakeholders in regard to the data analysis on encrypted data (ML use case) provided empirical data to understand their perspectives, and thus helped to identify key concerns and requirements. The results of our study show that the importance of data protection in eHealth is understood and valued by all stakeholders. Having differences in expertise among our stakeholders with medical, technical, and research backgrounds was significant for analyzing and identifying perceived privacy benefits and tradeoffs in our evaluation. Our results highlight that such differences in backgrounds could also impact the perception and trust in the claim that the data analysis on encrypted data is possible for protecting privacy without compromising data accuracy.

Assurance guarantees for the ML-based privacy enhancing tool’s privacy, accuracy, and capability to protect patients’ safety should be in place for establishing trust in the tool.

To address such perceptions and the correct understanding of tradeoffs, the communication and cooperation of eHealth stakeholders with diverse expertise could help in clarifying questions in regard to the accuracy of the technologies and medical safety of patients. Future research and practice could therefore consider involving a discussion among different stakeholders in the collaborative design and development processes.

Identified trust factors and elicited requirements are not only important for the PAPAYA project but can also be generalized to similar ML-based PETs for automated data analysis of medical data on cloud servers.

## Appendix

Multimedia Appendix 1 Guide for semistructured interview with stakeholders for the PAPAYA use case.

Multimedia Appendix 2 Simplified results from aggregated codes to themes and number of referenced interviews.

## Abbreviations

AI: artificial intelligence
ECG: electrocardiogram
eHealth: electronic health
GDPR: General Data Protection Regulation
HCI: human-computer interaction
ML: Machine Learning
PAPAYA: PlAtform for PrivAcY preserving data Analytics
PET: privacy-enhancing technology
PIA: privacy impact assessment
