# Supplementary material for: Machine Learning–Based Analysis of Encrypted Medical Data in the Cloud: Qualitative Study of Expert Stakeholders’ Perspectives
Source: JMIR Hum Factors. 2021 Sep 16;8(3):e21810. doi: 10.2196/21810 (PMC8485196; doi:10.2196/21810)
Supplement: Multimedia Appendix 1 [file humanfactors_v8i3e21810_app1.docx]

### Guide for Semi-structured Interview with stakeholders for PAPAYA use case

**Note: All text in blue are internal comments (instructions and motivation for the questions). Questions to be asked are in black, framed, and numbered. The three figures used were printed out, they correspond to the figures in the paper, we therefore illustrated where they stand in the guide.**

Research objectives of the interviews:

- Analyse the interviewee’s understanding, perception and trust about PAPAYA’s described tool and its medical use case.
- Based on this analysis: Elicit user requirements concerning: How the stakeholders should be introduced and informed about the PAPAYA’s platform and tools? Particularly, how to inform about the impact of PAPAYA on privacy and utility, so that they can understand the privacy benefits, potential risks and trust PAPAYA?
- (Elicit any requirements in regard to the use case set up, data flows)
- Opening: Welcome and short description of PAPAYA study, as well as outline of purpose of the Interview – explain the consent form, get approval, fill-in the form for demographic data with the interviewees.

1. **Introductory questions (background, privacy routines/experiences) – 5 min**

- For those employed by a healthcare organization:

| 1. What measures (if any) do you see for patient’s data privacy protection? | |
| --- | --- |
|  | 1.1. How do you manage day-to-day privacy of patient data in your practice / work? |
|  | 1.2. Are there occasions when you need to take special measures to protect your patient’s privacy, and how do you do  that? For example, perhaps there are investigations or test results that are particularly sensitive. |
| 2. Do you use pseudo-anonymization? | |
| 3. Do you come across data encryption in your practice? | |
| 4. Which do you think is used, and why? | |

In case that the terms are unknown, explain very briefly:

- e.g., encryption means that the information is encoded by scrambling it in a way that the information is hidden and can only be reconstructed (decrypted) later by an authorized party with the help of a secret key.
- Pseudo-anonymization means that all directly identifying data (such as the patient’s name) is replaced by a pseudonym.

| 5. Do you regard an ECG as a sensitive test? Or containing sensitive data? | |
| --- | --- |
|  | 5.1. How sensitive is ECG data in comparison to other data? |
|  | 5.2. Are there implications for the individual patient if there is a breach of confidentiality with their ECG data? |
| 6. Do you, or would you consider, engaging an external company to manage the security and privacy of your patients’ data? | |

1. **Use case introduction– 5 min**

<We introduce the use case with the help of a selected slide set >

Make clear that focus is on PAPAYA platform! <A printout based on Figure 1: Use case data analysis flow, is shown>

1. **Perception of privacy & trust (in privacy protection) – 10 min**

- How do doctors perceive the privacy advantages of doing data analytics only on encrypted data on the PAPAYA platform (– meaning that the patient’s medical data is only outsourced and processed by a 3rd party cloud provider in encrypted form and not accessible by that party in clear text?)

| 7. Would you in general have concerns to outsource (raw/unencrypted) ECG recording data to untrusted third parties? (even if pseudo-anonymized?) |
| --- |
| 8. Would you have any concerns if data analytics were conducted on non-encrypted form on an external (3rd party) cloud platform? |
| 9. Do you think that encryption is necessary regarding data analytics processed on the PAPAYA platform? |

- For this, we should test how they perceive different statements for the privacy-utility guarantees:
  - “The patient’s data will be analyzed in encrypted form for preventing that the patient’s personal data could leak to the PAPAYA analytics service – this form of analysis will not negatively impact the data quality.
  - “Further details on privacy protection:” [Here we show a comparison of the PIA results from the CNIL tool for an analysis service using PAPAYA and for one not using PAPAYA with some explanations)

| 10. How would your trust in PAPAYA be affected, if the following privacy statements are made: | | |
| --- | --- | --- |
|  | - “The patient’s data will be analyzed in encrypted form so that the patient’s private data cannot leak to the PAPAYA   analytics service – this form of analysis will not negatively impact the data quality”. | |
|  | - PIA done with the tool by the French data protection commissioner (CNIL), shows the risk reduction from a to b when   using PAPAYA. <we show corresponding figures: Figure 2: Risk assessment without PAPAYA (output from the CNIL PIA tool [38]) and Figure 3: Risk Assessment with PAPAYA (output from the CNIL PIA tool [38])> | |
|  | | 1. Privacy Risk Assessment for Data Analytics with and without PAPAYA |
|  | | 1. Privacy Risk Assessment for Data Analytics with PAPAYA |

Optional: Only for technically more skilled interviewees:

| 11. Would you have concerns if ECG data could be leaked in the case that PAPAYA’s crypto protection was actively “hacked” by  the cloud provider? | |
| --- | --- |
|  | 11.1. Would you require additional security measures to prevent such risks |

1. **Trust in PAPAYA (data quality) & Accountability – 5 min**

- Would stakeholders trust that the statement that data analytics could be completely conducted at the PAPAYA platform on encrypted data only, so that confidentiality and “integrity” (accuracy) of the medical data would be well protected?

| 12. Would you trust the results of data analytics on encrypted data (PAPAYA)? | | |
| --- | --- | --- |
|  | 12.1. | Why/why not? |
|  | 12.2. | Would you assume that analysis results on encrypted data would be accurate? |
|  | 12.3. | Would you prescribe a medical treatment based on the results? |

- Would stakeholders have any concerns in terms of accountability/liability when using PAPAYA?

| 13. Do you, as a stakeholder, have any say in the applications and infrastructure that are used? | | |
| --- | --- | --- |
|  | 13.1. Are you accountable for it? | |
|  | | 13.1.1. If yes: Would you have any concerns in terms of accountability/liability when using PAPAYA? |

1. **Informing Patients – 5 min**

- To what degree would they like to inform the patients about the level of privacy protection and any privacy-utility trade- offs (in particular in case that they are obtaining consent by the patients)?

14. To what degree would doctors like to inform the patients about privacy and integrity protection?

- Would medical stakeholders like to be prepared to answer also any potential questions by the patient concerning privacy protection?

14.1. Would you like to be prepared to answer patients’ questions regarding that (above mentioned)?

14. Would you like to know how patient’s data privacy is protected?
