# Supplementary material for: Machine Learning–Based Analysis of Encrypted Medical Data in the Cloud: Qualitative Study of Expert Stakeholders’ Perspectives
Source: JMIR Hum Factors. 2021 Sep 16;8(3):e21810. doi: 10.2196/21810 (PMC8485196; doi:10.2196/21810)
Supplement: Multimedia Appendix 2 [file humanfactors_v8i3e21810_app2.docx]

Simplified results from aggregated codes to themes and number of referenced interviews.

| Theme & subthemes | Aggregated high level codes | No. of referenced interviews |
| --- | --- | --- |
| **1.Contextual protection of the ECG data** | ECG protection as all medical protection | 14 |
|  | Not knowing what is needed technically | 5 |
|  | Pseudo-anonymization is accepted | 11 |
|  | More protection in communication and transfer of data | 4 |
|  | More protection against privacy leaks needed | 6 |
| **2. Conditional sensitivity of ECG data** | Legal sensitivity | 9 |
|  | Association to being medical data sensitivity | 13 |
|  | Association to biometric data sensitivity | 2 |
|  | Sensitive in combination with other data | 4 |
|  | Practically less sensitive than other data | 12 |
|  | How data are collected determines sensitivity | 2 |
| **3. Acceptance of using external services** | Overall acceptance but no 100% privacy guarantees | 10 |
|  | Acceptance of some known risks depending on benefits | 3 |
|  | Already use external services for value | 7 |
|  | Still preference of using internal services | 3 |
| **4. Data protection drawbacks** | Pseudo-anonymization not common in clinical use | 5 |
|  | Data protection not most important | 1 |
|  | Encryption is unseen | 10 |
|  | Encrypted data are protected better | 10 |
|  | Encrypted data concerns | 4 |
|  | Too much encryption issues | 2 |
|  | Data loss incidents | 2 |
| **5. Concerns of data analysis on encrypted data** | Algorithm concerns | 2 |
|  | Data accuracy concerns | 1 |
|  | Analysis on encrypted data skeptic | 5 |
|  | Missing technical knowledge | 1 |
|  | Missing medical knowledge | 1 |
|  | Technical plausibility doubts | 2 |
|  | Concerns of not having cardiologist guidance in the process | 3 |
| 5.1 Necessities for acceptance | Acceptance based on tests, proof and validation | 3 |
|  | Acceptance but require other data sources | 2 |
| **6. Communicating privacy and utility benefits and tradeoffs** | Technical plausibility concerns | 2 |
|  | Encryption concerns | 2 |
| 6.1 Information for trust | Trust depends on protection level | 1 |
|  | Trust depends on provider of technology | 1 |
|  | Trust depends on the tool | 2 |
|  | Trust depends on further information | 2 |
| 6.2 Reassurances for trust | Positive impact if PIA is done | 5 |
|  | More information needed overall | 1 |
| 6.3 PIA (privacy and utility benefits) assurances | Details needed about PIA | 3 |
|  | PIA testing and proofs needed | 5 |
|  | Certifications of PIA needed | 5 |
|  | Risk assessment needed | 1 |
| **7. Shared responsibility for patient privacy** | Patient’s privacy a shared responsibility | 8 |
|  | Researchers’ responsibility | 4 |
|  | Security team responsibility | 1 |
| **8. Informing patients on a higher level of abstraction** | Technical knowledge of details not needed | 8 |
|  | Information should be available | 14 |
|  | Delegation to leaflets or experts | 8 |
|  | Trust of organization is essential | 2 |
|  | Simple details is sufficient | 3 |
